# Sex‐Based Disparities in Index Cases of Familial Hypercholesterolemia in Vietnam: A Cross‐Sectional Study

**DOI:** 10.1002/hsr2.70579

**Published:** 2025-03-20

**Authors:** Ngoc‐Thanh Kim, Doan‐Loi Do, Mai‐Ngoc Thi Nguyen, Thanh‐Tung Le, Hong‐An Le, Thanh‐Huong Truong

**Affiliations:** ^1^ Department of Cardiology Hanoi Medical University Hanoi Vietnam; ^2^ Vietnam National Heart Institute, Bach Mai Hospital Hanoi Vietnam; ^3^ Faculty of Medicine Phenikaa University Hanoi Vietnam

**Keywords:** atherosclerotic cardiovascular disease, familial hypercholesterolemia, LDL‐C, statins

## Abstract

**Background and Aims:**

Familial hypercholesterolemia (FH) is a substantial contributor to the development of atherosclerotic cardiovascular disease. Therefore, the primary focus of our study was to examine sex‐based disparities in clinical signs, atherosclerotic status, lipid profiles, and treatment intensity among patients with FH from Vietnam.

**Methods:**

This retrospective cross‐sectional report analyzed the clinical profiles of 110 patients with FH from the Vietnam Familial Hypercholesterolemia (VINAFH) registry.

**Results:**

Among these patients, 47 (42.7%) were females, and 48 (43.6%) had mutant FH. Women were diagnosed with FH at a significantly later age than men. However, smoking and clinical signs suggestive of FH were observed more frequently in males than in females. Male patients exhibited a higher prevalence of premature coronary artery disease than females. No significant differences in plasma total cholesterol and low‐density lipoprotein cholesterol (LDL‐C) levels were observed between sexes. In males, the areas under the curve (AUC) for plasma LDL‐C levels were 0.83, with a cut‐off value of 6.11 mmol/L (sensitivity, 79.4%; specificity, 89.7%). In females, the AUC for plasma LDL‐C levels was 0.72, with a cut‐off value of 6.9 mmol/L (sensitivity, 57.1%; specificity, 93.9%). Statins were prescribed to most patients (93.6%), with a higher proportion of men than women receiving high‐intensity statin therapy.

**Conclusion:**

Our findings suggested that in Vietnam, FH is diagnosed later in women, whereas men are more likely to smoke and have atherosclerotic cardiovascular disease. Treatment intensity in female patients with FH was lower than that in male patients, despite statin prescription.

## Summary

- Women are less likely to receive high‐potency lipid‐lowering regimens for achieving satisfactory plasma LDL‐C levels compared with men.
- FH is diagnosed later, and treatment intensity is lower in women than in men in Vietnam.
- This study's findings highlight the need for future programs that provide early screening, diagnosis, and treatment for FH.

## 1 Introduction

Familial hypercholesterolemia (FH) is a genetic condition characterized by lifelong elevated levels of plasma total cholesterol and low‐density lipoprotein cholesterol (LDL‐C). It is estimated that 1 in 250 individuals is affected by FH; however, many patients are underdiagnosed and undertreated [1], as patients with FH may remain asymptomatic until they experience atherosclerosis. Women with FH are at higher risk of cardiovascular diseases owing to premature atherosclerosis; even after an ischemic coronary or stroke event, women have a 10% lower likelihood of being diagnosed with FH than men [2]. However, intensive and prompt treatment with lipid‐lowering medications can significantly reduce the risk of cardiovascular disease in both women and men with FH [3].

Recent reports have highlighted the underutilization and suboptimal management of lipid‐lowering medications in women with FH compared with their male counterparts. This is primarily a result of concerns related to pregnancy and challenges to achieving LDL‐C targets [4]. To enhance outcomes and diminish sex‐related inequities, it is crucial to incorporate women in registries and trials investigating FH and the effects of lipid‐lowering therapies [5]. The latest findings from the European Atherosclerosis Society‐Familial Hypercholesterolemia Studies Collaboration (EAS‐FHSC) registry also elucidated sex‐specific differences in FH. Despite similar untreated plasma LDL‐C levels and prevalence of cardiovascular risk factors, the incidence rate of coronary artery disease in women was half that of men. Moreover, on average, women are diagnosed with FH at a later age than men [6]. Sex differences were also found in therapy, with women being less likely to receive higher potency lipid‐lowering regimens and achieve LDL‐C goals. Concerns regarding the treatment of women of childbearing potential may contribute to variations in the use of lipid‐lowering medications.

The unexplained lower achievement of LDL‐C targets in women compared with men remains a persistent issue that may be attributed to various factors, including barriers in treatment, medication adherence, and socio‐cultural‐economic factors [7]. However, analysis of sex disparities in diagnosis and treatment for FH in developing countries such as Vietnam remains limited. Nonetheless, FH represents a significant public health concern regarding cardiovascular disease. A recent study reported that the prevalence of potential FH in Vietnamese patients with premature acute myocardial infarction is as high as 14.7%. Patients with FH have significantly higher plasma LDL‐C levels than those without FH. Additionally, patients with FH exhibited greater severity of coronary artery lesions than their counterparts [8]. A large study involving 751 patients with homozygous FH (HoFH) from 38 countries, including 353 patients across 18 lower‐upper middle‐income nations, indicated that HoFH is associated with a markedly elevated risk of premature atherosclerotic cardiovascular disease (ASCVD) without discernible sex differences [9]. These findings suggest the importance of early diagnosis and intervention for both sexes. To address this concern, we aimed to confirm sex disparities in the clinical manifestations and treatment of patients with FH in Vietnam. This study will provide important insights into improving clinical management and reducing outcome disparities for FH.

## 2 Methods

### 2.1 Study Design and Population

We conducted this cross‐sectional study based on the Vietnam Familial Hypercholesterolemia Registry [10, 11] and included FH index cases from December 2020 to December 2023. The study was conducted in accordance with the guidelines of the Declaration of Helsinki and was approved by the Ethics Committee of Hanoi Medical University (number: 39/GCN‐HĐĐĐNCYSH‐ĐHYHN, date of approval: April 19, 2021). Informed consent was obtained from all participants involved in the study. For minors with FH younger than 18 years of age, their parent and/or legal authorized representatives provided written informed consent after receiving an explanation of the study objective and procedures.

### 2.2 Inclusion Criteria

We enrolled phenotypic FH index cases detected by opportunistic screening in patients with premature ASCVD and/or hypercholesterolemia. In adults, phenotypic FH was confirmed on the basis of the Dutch Lipid Clinic Network (DLCN) criteria ≥ 3 points [12, 13, 14]. In children aged < 18 years, phenotypic FH was diagnosed on the basis of the presence of an LDL‐C level consistent with that in FH in addition to a family history of premature ASCVD, and/or a high baseline cholesterol level in one parent, and/or the presence of an FH‐causing mutation [13]. All patients underwent genetic testing for *LDLR/APOB/PCSK9* and were classified as HoFH, carrying the same mutations in both alleles of FH genes, and heterozygous FH (HeFH), carrying only one mutation in the alleles of FH genes, and compound HeFH, carrying two mutations in the same FH gene, and double HeFH, carrying one mutation in each of two FH genes, or no mutant FH, not carrying any mutation in the alleles of FH genes.

### 2.3 Exclusion Criteria

Exclusion criteria for participants included FH relatives of index cases in this study and individuals with known medical conditions causing second hyperlipidemia, such as hypothyroidism, nephrotic syndrome, cholestasis, and hypopituitarism.

### 2.4 Variables

Medical, family history, clinical data including age and sex, and lipid profile were collected for each patient, based on the DLCN criteria. Age groups were defined as follows: children and young adults, < 35 years; adults, 35–44 years; middle‐aged, 45–65 years; and older adults, > 65 years. Clinical signs suggestive of FH, such as xanthomas and arcus corneae, were also recorded. For each patient, information on ASCVD (coronary artery disease, ischemic stroke, carotid stenosis, peripheral artery disease, (supra)valvular aortic stenosis) and family history (hypercholesterolemia, stroke, and coronary artery disease) were collected. Premature ASCVD was characterized as an initial clinical manifestation before the ages of 55 and 60 years in men and women, respectively. Additionally, we collected information on cardiovascular risk factors such as hypertension, diabetes, smoking status, overweight and/or obesity. Maximum plasma levels of total cholesterol, triglyceride, LDL‐C, and HDL‐C were measured for each patient, with their reference values in Vietnam as < 5.2, < 1.7, 2.59, and ≥ 1.55 mmol/L, respectively. Ongoing lipid‐lowering therapies, such as the types and dosages of statin medications for each patient, were also recorded.

### 2.5 Statistical Analysis

The collected data were processed and analyzed using SPSS software (version 26.0; IBM Corp., Armonk, New York, USA). Quantitative data are presented as mean ± standard deviation (95% confidence interval [CI]) for normally distributed continuous variables, or median (interquartile range) for non‐normally distributed continuous variables. Nominal variables are described as absolute number (*n*) and percentage. Qualitative data as nominal variables are presented as absolute number (*n*) with frequency or percentage (%). For intergroup comparisons with nominal variables, chi‐square or Fisher exact tests (if the cell counts were < 5) were employed. The t‐tests were performed for mean comparisons in normally distributed continuous variables, whereas differences in non‐normally distributed variables were assessed using the Mann–Whitney *U*‐test. Receiver operating characteristic curve analysis was conducted to determine the optimal cut‐off points, sensitivity, specificity, and area under the curve (AUC) for plasma levels of total cholesterol and LDL‐C in detecting *LDLR/APOB/PCSK9* mutations. In all analyses, statistical significance was defined as a two‐tailed *p*‐value < 0.05.

## 3 Results

In total, this study included 110 patients with FH (63 men and 47 women). Detailed descriptions of clinical characteristics and lipid levels according to sex are presented in Table 1. Female patients were diagnosed at a significantly older age than male patients (61 vs. 50 years; *p* = 0.001). Most female patients were diagnosed late, with one‐quarter of patients diagnosed before the age of 45 years. Smoking was more common among male patients, with over two‐thirds being smokers and no female patients reporting smoking (*p* < 0.001). Hypertension was common in more than one‐third of patients with FH, and there were no significant differences between sexes (40.4% vs. 34.9%). Diabetes and overweight or obesity were common among patients (5.5% and 14.5%, respectively) and similar between sexes. Clinical signs indicative of FH, such as arcus corneae before 45 years of age, xanthomas, and familial history of coronary artery disease and hypercholesterolemia, were present among patients. Male patients with FH had a significantly higher prevalence of coronary artery disease and premature coronary artery disease than female patients with FH (50.8% vs. 14.9%, *p* < 0.001, and 55.5% vs. 27.7%, *p* = 0.004, respectively; Table 1). Male patients with FH also tended to have a higher incidence of stroke, carotid stenosis, or both than female patients with FH, although the difference was not statistically significant. Notably, women and men had similar plasma TC and LDL‐C levels.

**Table 1:** Characteristics of patients with familial hypercholesterolemia.

Characteristics	Total (*n* = 110)	Women (*n* = 47)	Men (*n* = 63)	*p* value
Age at diagnosis years, median [IQR]	54 [17.3]	61 [11.0]	50 [14.0]	***0.001***
Classification of age at diagnosis				
Children and young adult, *n* (%)	10 (9.1)	3 (6.4)	7 (11.1)	*0.1*
Adult, *n* (%)	17 (15.5)	4 (8.5)	13 (20.6)	
Middle‐age, *n* (%)	71 (64.5)	32 (68.1)	39 (61.9)	
Old‐age, *n* (%)	12 (10.9)	8 (17.0)	4 (6.3)	
Primary cardiovascular risk factors				
Smoking (*n*, %)	48 (43.6)	0	48 (76.2)	***< 0.001***
Hypertension (*n*, %)	41 (37.3)	19 (40.4)	22 (34.9)	*0.56*
Diabetes (*n*, %)	6 (5.5)	3 (6.4)	3 (4.8)	*> 0.99*
Overweight or obesity (*n*, %)	16 (14.5)	6 (12.7)	10 (15.9)	*0.648*
Clinical signs				
Arcus cornealis before 45 years, *n* (%)	13 (11.8%)	1 (2.1)	12 (19.0)	***0.007***
Xanthomas, *n* (%)	20 (18.2%)	7 (14.9)	13 (20.6)	*0.44*
Ischemic stroke and/or carotid stenosis	18 (16.4)	6 (12.8)	12 (19.0)	*0.38*
Coronary artery disease	48 (43.6)	13 (27.7)	35 (55.6)	***0.004***
Premature coronary artery disease	39 (35.5)	7 (14.9)	32 (50.8)	***< 0.001***
Family history				
Hypercholesterolemia, *n* (%)	40 (36.4)	16 (34.0)	24 (38.1)	*0.66*
Coronary artery disease, *n* (%)	21 (19.1)	6 (12.8)	15 (23.8)	*0.15*
Ischemic stroke, *n* (%)	19 (17.3)	9 (19.1)	10 (15.9)	*0.65*
Lipid profile (mmol/L)				
Total cholesterol, mean [IQR]	8.21 [3.26]	7.88 [1.69]	8.66 [3.58]	*0.18*
Triglyceride, mean [IQR]	2.03 [1.93]	2.05 [1.57]	2.03 [2.13]	*0.29*
HDL‐C, mean [IQR]	1.24 [0.49]	1.42 [0.48]	1.15 [0.53]	*0.05*
LDL‐C, mean [IQR]	5.69 [2.61]	5.52 [1.26]	5.96 [3.35]	*0.31*

*Note: p*‐values with bold and italics are statistical significance.

Abbreviations: HDL‐C, high‐density lipoprotein cholesterol; IQR, interquartile range; LDL‐C, low‐density lipoprotein cholesterol.

**Figure 1:**
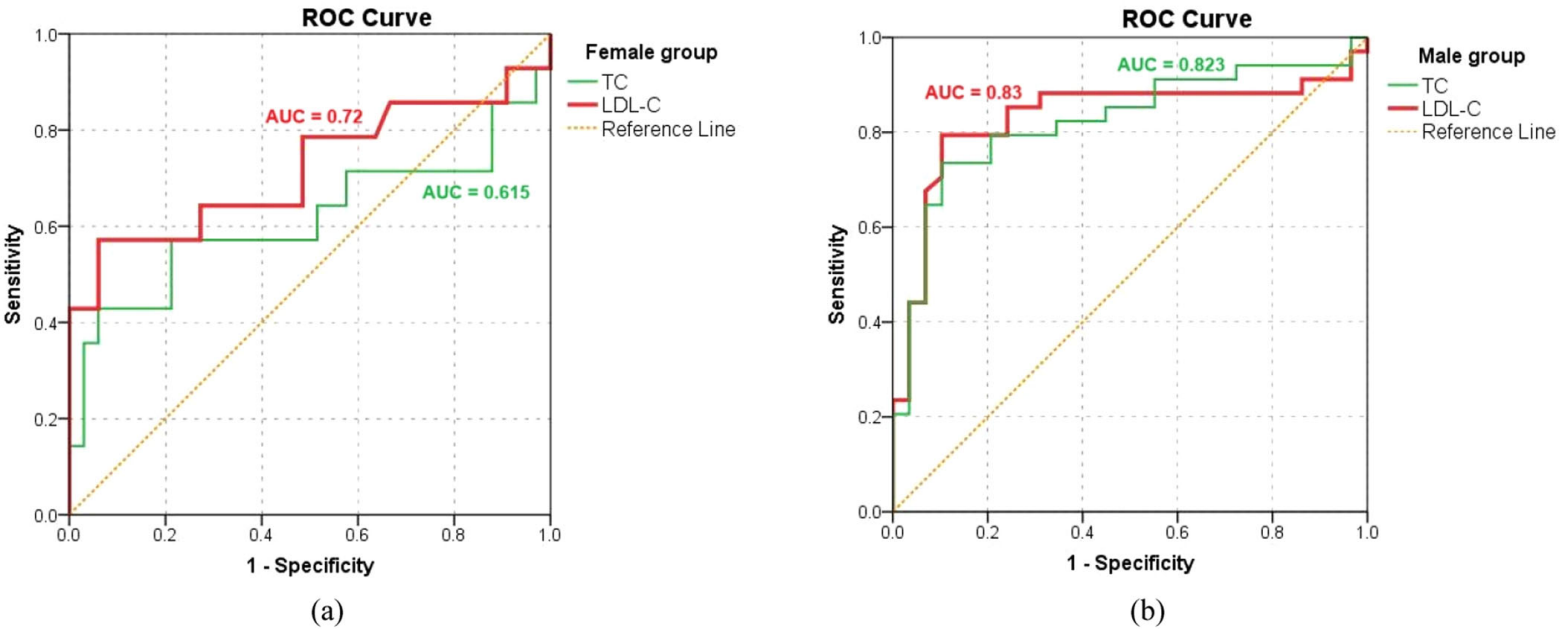
Receiver operating characteristic curves for positive familial hypercholesterolemia genetic testing of cholesterol levels. AUC, area under the curve; LDL‐C, low‐density lipoprotein cholesterol; ROC, receiver operating characteristic; TC, total cholesterol.

In this study, the mutation rate of *LDLR/APOB/PCSK9* was 43.6% (*n* = 48/110). Figure 1 shows the AUC for positive FH genetic testing results. In males, the AUC for the maximum plasma total cholesterol level was 0.823 (95% CI, 0.715–0.93; *p* < 0.001), with a cut‐off value of 8.83 mmol/L (sensitivity, 73.5%; specificity, 89.7%). The AUC for the maximum LDL‐C level was 0.83 (95% CI, 0.718–0.943; *p* < 0.001) with a cut‐off value of 6.11 mmol/L (sensitivity, 74.4%; specificity, 89.7%). In females, the AUC for the maximum plasma total cholesterol level was 0.615 (95% CI, 0.403–0.826; *p* = 0.22). The AUC for the maximum LDL‐C level was 0.72 (95% CI, 0.529–0.91; *p* = 0.018) with a cut‐off value of 6.9 mmol/L (sensitivity, 57.1%; specificity, 93.9%).

Table 2 displays the status of lipid‐lowering therapy. A total of 93.6% of patients used statins with a significant difference observed between sexes (*p* = 0.02). The prescription rate of high‐intensity statins was three times lower in females than that recorded in males (10.6% vs. 36.5%).

**Table 2:** Lipid‐lowering therapy in patients with familial hypercholesterolemia.

Lipid‐lowering therapy	Total (*n* = 110)	Women (*n* = 47)		Men (*n* = 63)	*p* value
High‐intensity statin, *n* (%)	*28 (25.5)*	*5 (10.6)*		*23 (36.5)*	***0.02***
Rosuvastatin 20 mg	25 (22.7)	5 (10.6)		20 (31.7)	
Atorvastatin 40 mg	3 (2.7)	0		3 (4.8)	
Moderate‐intensity statin, *n* (%)	*66 (60.0)*	*34 (72.3)*		*32 (50.8)*	
Rosuvastatin 5 mg	5 (4.5)	4 (8.5)		1 (1.6)	
Rosuvastatin 10 mg	23 (20.9)	8 (17)		15 (23.8)	
Atorvastatin 10 mg	10 (9.1)	6 (12.8)		4 (6.3)	
Atorvastatin 20 mg	15 (13.6)	8 (7.3)		7 (11.1)	
Pitavastatin 2 mg	11 (10.0)	6 (12.8)		5 (7.9)	
Simvastatin 40 mg	1 (0.9)	1 (2.1)		0	
Simvastatin 20 mg	1 (0.9)	1 (2.1)		0	
Low‐intensity statin, *n* (%)	*9 (8.2)*	*5 (10.6)*		*4 (6.3)*	
Atorvastatin 5 mg	1 (0.9)	0		1 (1.6)	
Pitavastatin 1 mg	6 (5.5)	3 (6.4)		3 (4.8)	
Pravastatin 20 mg	1 (0.9)	1 (2.1)		0	
Pravastatin 10 mg	1 (0.9)	1 (2.1)		0	
No statin use, *n* (%)	*7 (6.4)*		3 *(6.4)*	*4 (6.3)*	

*Note: p*‐values with bold and italics are statistical significance.

## 4 Discussion

Our study is the first report from Vietnam on sex differences in FH. Its results reinforce the findings observed in other developing countries and emphasize the need for future programs aimed at early screening, diagnosis, and treatment. Generally, patients with FH tend to experience delayed diagnosis, particularly those who are women. High plasma cholesterol levels, which were similar in both sexes, serve as a primary diagnostic indicator for FH screening including predicting positive mutations. Male patients had high rates of cardiovascular risk factors such as hypertension and smoking as well as premature coronary artery disease, suggesting the need for targeted counseling and intervention programs. Conversely, female patients with FH seemed to be undertreated with lipid‐lowering therapy.

Our study highlighted that FH diagnosis often occurs late—typically after the age of 40 years, revealing the need for early screening programs in Vietnam. This late diagnosis trend is not unique to Vietnam and is prevalent worldwide. According to the largest global perspective involving over 61,000 patients with FH from 56 countries in the EAS‐FHSC, the average age at FH diagnosis was 44.4 years, with 40.2% detected before 40 years of age [6]. This delayed diagnosis reflects a gap in FH screening and care. Regarding genetic metabolism, untreated FH contributes to the burden of ASCVD related to lifelong exposure to elevated cholesterol levels and delayed lipid‐lowering interventions. Therefore, clinical recommendations and consensus on FH emphasize the global need to improve early screening, diagnosis, and treatment starting as early as adolescence [15]. Our study also observed a notable delay in FH diagnosis among women compared with men, with a delay of up to 11 years. This delay is even more severe than what has been reported in recent reports indicating female individuals are diagnosed with FH approximately 4 years later than men, potentially resulting in an estimated 16‐year lifespan reduction for affected patients [4]. In our study, the reasons for this delay in diagnosis are likely multifactorial and not solely owing to more severe symptoms and higher rates of ASCVD in men compared with women, which is consistent with previous data [16]. Our study found that LDL‐C levels and the prevalence of xanthomas were comparable between sexes. Xanthomas is a characteristic feature of FH, presenting as yellowish plaques under the skin primarily around the joints, or as yellowish streaks in the eyelids or tendons [17]. Additionally, our study revealed that premature arcus corneae was more common in male patients with FH than in their female counterparts, which may be related to an increased risk of cardiovascular events. This finding aligns with recent research indicating that arcus corneae is a predictor of cardiovascular disease, independent of total cholesterol and HDL‐C plasma levels and smoking status [18].

Patients with FH bear a substantial lifelong burden of high cholesterol and atherosclerotic risk, which requires early intervention. Additionally, they face additional cardiovascular risk factors that can exacerbate atherosclerosis progression, contributing to increased future cardiovascular events. In this regard, we verified that traditional cardiovascular risk factors such as smoking, hypertension, diabetes, and overweight or obesity were common in Vietnamese patients with FH. According to the EAS‐FHSC registry, the prevalences of diabetes, hypertension, and smoking in FH patients were 5%, 19.2%, and 23.5%, respectively [6]. The hypertension rate in Vietnam is increasing, with recent reports indicating a prevalence ranging from 19% to 31% [19]. In our study cohort, both sexes exhibited higher rates of hypertension than the general population, with 34.9% in men and 40.4% in women. Other studies in patients with FH have also reported common concomitant cardiovascular risk factors. Data from the Hellenic Familial Hypercholesterolemia Registry presented prevalences of 29.7% and 7.9% for hypertension and diabetes in adults with FH, respectively [20]. Smoking, a leading risk factor of cardiovascular disease in Vietnam and across Asia, is strongly sex‐linked, with approximately 45.3% of men being current smokers, while smoking is rare among women [21]. In our study, no women with FH smoked, whereas more than three‐quarters of men with FH were smokers. In the GIRaFH study, cardiovascular atherosclerotic events increased in smokers with FH compared with nonsmokers, with an estimated risk ratio of 2.1; however, this risk decreased after smoking cessation. In the model with the highest likelihood value, the risk reduction of smoking after cessation follows a linear trend over time, taking nearly 10 years for the excess risk to reduce to zero [22]. Therefore, to better manage FH in Vietnam, smoking cessation should be considered in male patients to mitigate their cardiovascular risk. Our results suggest that behavioral aspects and cardiovascular factors must be considered in guiding health policies for patients with FH. In addition to lipid‐lowering therapies, early adoption of healthy lifestyles should be prioritized as the first step in counseling and prevention of cardiovascular disease in these patients [23].

In our study, nearly half of the FH index cases had coronary artery disease. Many of these cases experienced cardiovascular events at a younger age, which can likely be attributed to the delay in diagnosis and treatment. The prevalence of premature coronary artery disease was higher in men than in women; ischemic stroke and carotid stenosis were also common but had a lower prevalence than coronary artery disease. According to 2022 statistics from the American Heart Association, the prevalence of most traditional cardiovascular risk factors in women before menopause tended to be lower than that in men [24]. However, the differences may become more apparent in underdiagnosed and undertreated patients with FH. Similarly, the EAS‐FHSC registry has reported a high prevalence of coronary disease, with the rate being two times lower in female patients than in males [6]. In a recent analysis of patients with HoFH, the prevalence of myocardial infarction was also higher in males than in females (16.3% vs. 8%) [9]. In clinical scores used to detect individuals suspected of having FH, personal medical history, family history of hypercholesterolemia, and ASCVD carry significant weight. This further reinforces selective screening strategies for FH, focusing on individuals with premature ASCVD in Vietnam and other developing countries where resources are limited for universal screening, aligning with current guidelines [12, 13, 14, 15]. In addition, a significant number of patients with FH in our study had a family history of hypercholesterolemia, coronary artery disease, and ischemic stroke. This suggests that FH follows a dominant inheritance pattern. Therefore, it is highly recommended to conduct selective screening for individuals with a family history of hypercholesterolemia or early‐onset cardiovascular disease. Using cascade screening methods can be extremely effective in identifying patients with FH to facilitate treatment initiation before they experience any complications [15, 25, 26].

In addition, our study showed elevated plasma levels of total cholesterol and LDL‐C in patients with FH but no differences between both sexes; however, plasma levels of HDL‐C and triglyceride remained within normal ranges, similar to those in the EAS‐FHSC registry [6]. FH is an autosomal dominant inheritance disorder caused by genetic mutations that result in lifelong LDL‐C elevation. Recent screening data from the Copenhagen General Population Study reported that approximately 1 in 200 individuals have FH, defined by LDL‐C plasma levels > 4.4 mmol/L. Current guidelines recommend selective screening for FH in individuals with hypercholesterolemia [15, 27]. Using a cut‐off threshold simplifies FH screening, preventing the need for comprehensive clinical information or genetic testing, which can help prevent cardiovascular complications. The US‐MedPed criteria, using cut‐off values of total cholesterol adjusted the patient age range and relative specificity, helped to identify individuals with FH in the population [28]. While easy to use, the US‐MedPed criteria are not precisely determined, with a sensitivity of 25.3% in an Asian population [29]. The cut‐off threshold for FH varies due to factors such as genetics, sex, and race, requiring each country to develop its diagnostic threshold for the initial screening program [30, 31]. In this study, we identified the maximum LDL‐C plasma levels with cut‐off values of 6.11 mmol/L for men and 6.9 mmol/L for women, which could effectively predict the presence of FH‐associated mutations in Vietnam. These findings suggest that both plasma levels of total cholesterol and LDL‐C can be used as indicators for genetic testing in patients with FH, with higher sensitivity and specificity observed in males than in females.

Additionally, we found that lipid‐lowering therapy in female patients tended to be suboptimal compared with that in male patients because of the lower rate of high‐intensity statin use. This highlights the importance of individualizing treatment approaches and considering sex‐specific factors when optimizing lipid‐lowering treatments for women with FH. Recent studies have reported sex‐based disparities in statin administration, revealing that women eligible for statins are less likely to receive any medications or the recommended intensity of statins than men [32]. Previous studies have shown that women have a lower prescription rate for high‐intensity statins than men, with a difference of 31% [33]. Some hypotheses regarding this phenomenon include concerns about medication use in women, particularly during pregnancy or breastfeeding [4]. However, this issue of lipid‐lowering therapy undertreatment in women with FH remains a challenge not only in developing countries like Vietnam but also in high‐income countries. For example, in Spain, only 44% of women with FH receive high‐intensity lipid‐lowering therapy compared with approximately 60% of men. Consequently, these women demonstrated lower adherence to lipid‐lowering therapy and a lower probability of achieving LDL‐C targets than men, particularly in cases of secondary cardiovascular prevention [7]. Prolonged undertreatment may heighten the risk of atherosclerotic cardiovascular events among women with FH.

This study has some limitations. Despite making significant efforts to report the first data on patients with FH in Vietnam, the study faced limitations due to limited resources, particularly funding for genetic testing. Consequently, the study included a small sample size of 110 patients, which may affect the generalizability of the findings. Furthermore, our data did not include detailed information on atherosclerotic imaging, as well as the treatment and follow‐up response of lipid‐lowering therapy in relation to sex‐based disparities.

## 5 Conclusions

This study provides additional insights into managing FH based on sex differences in Vietnam and emphasizes the importance of early diagnosis, tailored management strategies, and consideration of sex‐specific factors in managing FH. Understanding FH in women is essential for improving treatment outcomes and reducing the burden of cardiovascular disease in this population. Therefore, future studies should increase the sample size to improve the generalizability of the findings and provide more robust statistical power. Additionally, we aim to collect more comprehensive information about the diagnosis and treatment of patients with FH, including their response to lipid‐lowering medications, the relationship between lipid‐lowering therapy and specific mutations, and long‐term cardiovascular outcomes. By doing so, a more detailed assessment of sex‐based differences in the management of FH can be achieved, providing valuable insights for the development of appropriate strategies to enhance the quality of care and prevention of ASCVD for patients with FH in Vietnam.

## Author Contributions

**Ngoc‐Thanh Kim:** conceptualization, investigation, funding acquisition, writing – original draft, methodology, validation, visualization, writing – review and editing, formal analysis, project administration, data curation, software, resources. **Doan‐Loi Do:** conceptualization, investigation, writing – original draft, methodology, writing – review and editing, funding acquisition. **Mai‐Ngoc Thi Nguyen:** conceptualization, investigation, funding acquisition, writing – original draft, methodology, writing – review and editing. **Thanh‐Tung Le:** conceptualization, investigation, writing – review and editing, writing – original draft, methodology. **Hong‐An Le:** writing – original draft, investigation, conceptualization, methodology, writing – review and editing. **Thanh‐Huong Truong:** conceptualization, investigation, funding acquisition, writing – original draft, methodology, validation, visualization, writing – review and editing, supervision, project administration, resources, formal analysis, software, data curation.

## Conflicts of Interest

The authors declare no conflicts of interest.

## Transparency Statement

The lead author, Thanh‐Huong Truong, affirms that this manuscript is an honest, accurate, and transparent account of the study being reported; that no important aspects of the study have been omitted; and that any discrepancies from the study as planned (and if relevant, registered) have been explained.

## Supporting information

Supporting information.

## Data Availability

The authors confirm that the data supporting the findings of this study are available within the article and its supplementary materials; further inquiries should be directed to the corresponding author.
